# Advanced Nodular Thin Dense Chromium Coating: Superior Corrosion Resistance

**DOI:** 10.1021/acsami.4c19897

**Published:** 2025-01-22

**Authors:** Ehsan Rahimi, Thijs Nijdam, Adwait Jahagirdar, Esteban Broitman, Arjan Mol

**Affiliations:** † Department of Materials Science and Engineering, Delft University of Technology, Mekelweg 2, 2628 CD Delft, The Netherlands; ‡ SKF Research & Technology Development, 3992 AE Houten, The Netherlands

**Keywords:** Thin dense chromium, Electroplated coating, Near-nanocrystalline structure, Compact bilayer oxide, Corrosion protection

## Abstract

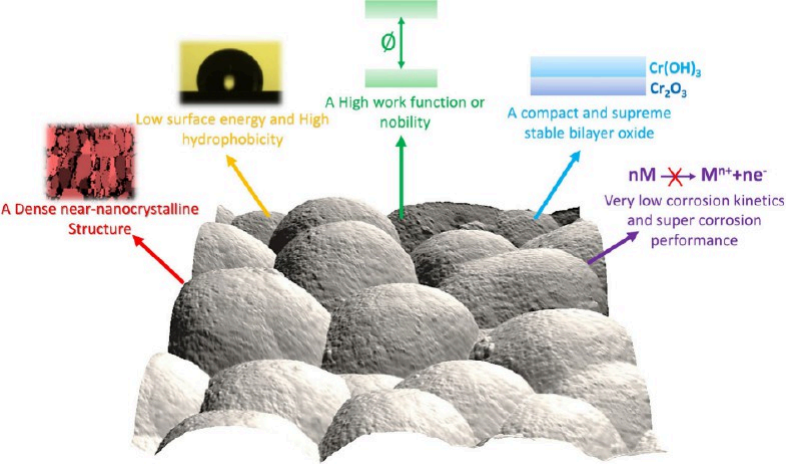

Chromium-based functional coatings (CFCs) are widely recognized for their outstanding wear and corrosion resistance across diverse industrial sectors. However, despite advancements in deposition techniques and microstructural enhancements, many contemporary CFCs remain vulnerable to degradation in highly corrosive environments. For the first time, this research delivers a thorough characterization of the corrosion resistance of advanced CFCs, focusing on the performance of a 5 μm thin dense chromium (TDC) coating. These TDCs exhibit a distinctive, uniform nodular microstructure, characterized by approximately 3.6 μm nodules composed of defect-free near-nanocrystalline grains (227 ± 75 nm) plus enhanced electrochemical nobility. This structure promotes the rapid formation of a stable, dense bilayer oxide, resulting in a remarkably low corrosion susceptibility, effectively impeding both charge transfer and mass transport, particularly the diffusion of Cl^–^ ions. Furthermore, the coating sustains an exceptionally high polarization resistance over extended exposure times in aqueous NaCl electrolyte. These findings offer critical insights into the design of CFCs optimized for extreme environmental durability.

## 1 Introduction

Preventing the corrosion of metallic materials, especially steel in demanding and corrosive environments, remains a crucial concern for various structural and functional applications.^1,2^ If corrosion is not properly managed, this may result in material failure, severe safety hazards as well as high economic and detrimental environmental impacts.^3^ Advanced surface protection and functionalization technologies using noble metals are essential for substantially enhancing the service performance and extending the lifespan of most electrochemically active materials.^4,5^ The use of chromium-based coatings have long been regarded as an exceptionally well-performing solution in protective strategies for applications in need of outstanding wear and corrosion resistance.^6^ The chromium passive film bilayer structure, consisting of a compact and thin Cr_2_O_3_ inner layer and an outer Cr(OH)_3_ layer, forms spontaneously and effectively obstructs aggressive ions (e.g., Cl^–^) from initiating localized corrosion.^7^ Extensive research has focused on enhancing chromium electroplating technologies by optimizing microstructural features (such as crystalline size, structure, morphology, porosity, and cracking),^8,9^ as well as improving mechanical properties (including hardness and wear resistance)^6,10^ and chemical stability (ensuring both short-term and long-term corrosion durability).^11,12^ Additionally, the incorporation of elements or microparticles like Cr–P,^13^ Cr–C,^14^ MoO_2_,^15^ TiO_2_,^15^ WC,^16^ and Cr–TiCN^17^ into chromium coatings has proven to significantly improve mechanical properties and reinforce their corrosion resistance.

However, a noteworthy drawback of many chromium coatings, whether in industrial hard chrome or decorative chrome, is the occurrence of defects like pinholes, microcracks, and, more critically, macrocracks.^18,19^ These defects act as active sites that accelerate the initiation of localized corrosion, such as pitting and crevice corrosion, where reduction reactions occur on coated regions, leading to the rapid anodic dissolution of the highly electrochemically active underlying metals.^13^ Effective control of grain size (usually fine grain), internal stress, and crack patterns in the microstructure of developed chromium-electroplated coatings plays a crucial role in improving their corrosion resistance.^8,12,13,19^ A thin dense chromium (TDC) coating, created through a specialized electroplating process, can effectively overcome all these challenges. SKF Research, Technology, and Development (RTD) has pioneered a proprietary electrodeposition process for advanced TDC coating, utilizing an innovative, in-house technique.^20^ This approach yields a nearly crack-free deposit with a nodular surface texture, in contrast to the microcracked structure commonly seen in most conventional hard chrome plating. According to the authors’ elaborate literature review, no further published research concerning the detailed microstructural characteristics, mechanical, and corrosion behavior of TDC coating has been documented in the literature.

In light of that, this research systematically explores the impact of TDC’s hierarchical nodular surface texture and dense near-nanocrystalline structures on the corrosion behavior in a 3.5% NaCl solution, an aggressive corrosive medium. By employing a comprehensive array of advanced techniques, including scanning electron microscopy and energy dispersive X-ray spectrometry (SEM-EDXS), atomic force microscopy (AFM), scanning Kelvin probe force microscopy (SKPFM), Electron backscatter diffraction (EBSD), X-ray photoelectron spectroscopy (XPS), and AC/DC multielectrochemical analyses, we strive to uncover intricate details of the near-nanocrystalline structure and orientations, morphological, electrochemical behavior, local surface electronic properties, and the evolution of complex thin oxide films during the corrosion process at micro and nanoscale. The results reveal that the near-nanocrystalline structure of TDC coatings significantly enhances their resistance to chloride-induced degradation, making them highly durable in corrosive environments. These findings provide crucial insights for optimizing chromium-based coatings for demanding applications in aerospace, automotive, and marine industries, where performance under extreme conditions is paramount. Thus, this work represents a significant advancement in protective coating technologies, with broad implications for industrial applications.

## 2 Experimental Section

### 2.1 Materials

The experimental electroplating information described here is an extension of our earlier research.^21^ This study employed thin dense chromium (TDC) and hard chromium coatings supplied by SKF, which were electrodeposited onto substrates made from 52100-bearing steel. The steel composition includes 0.98–1.10% carbon, 1.30–1.60% chromium, 0.15–0.30% silicon, and 0.25–0.45% manganese, with trace amounts of sulfur (≤0.025%) and phosphorus (≤0.025%). Both coatings processes commenced with degreasing the workpieces in a heated alkaline solution, followed by surface preparation through etching or gentle abrasive powder blasting. The arithmetic average roughness (*R*_a_) of carbon steel before the electroplating process was measured at 150 ± 30 nm. Subsequently, the components were submerged in a chromium acid electrolyte (Cr(VI)) solution (included for both coatings).^21,22^ However, potassium dichromate was utilized as a catalyst during the TDC electroplating process. This process facilitated the deposition of a thin chromium layer with the desired surface properties by employing a lower current density and reducing the coating duration. The procedure for both coatings concluded with cleaning and preservation steps to ensure the coating’s integrity and durability. The TDC coating is specifically engineered to enhance hardness and wear resistance. Its dense, microcrack-free microstructure provides excellent corrosion performance, while its tailored surface topography influences frictional behavior. These characteristics make TDC coatings particularly well-suited for applications requiring precise friction control. In bearing applications, the coating thickness is carefully regulated to approximately 5 μm, ensuring optimal surface functionality and performance.

### 2.2 Characterization

#### SEM, EDXS, and EBSD

The surface morphology and μstructure analyses were accomplished using a scanning electron microscope (Teneo, FEI, 5 kV, 0.4 nA, and a working distance of 10 mm) with secondary electron (SE) and backscatter electron (BSE) detectors. Electron backscatter diffraction (EBSD) signals of cross-sectional TDC coating were obtained at 15 kV, 6.4 nA, a scanning rate of 40 nm, and a working distance of 10 mm. For EBSD analysis, the TDC coating was first mechanically polished using a 3 μm alumina slurry, followed by final polishing with OPS (0.25 μm agglomerated particles) and OPU (0.04 μm agglomerated particles).

#### AFM and SKPFM

To examine the nanosurface topography and electronic surface potential evolution of TDC coating before and after exposure to aggressive 3.5% NaCl, AFM coupled with SKPFM measurements were performed. AFM and SKPFM mappings were performed using Bruker Dimension Edge Instrumnets with an antimony (n) doped silicon pyramid single crystal tip, coated with PtIr5 (SCM-Pit-V2 probe, tip radius, and height were 25 nm and 10–15 μm, respectively). The surface potential maps were recorded in dual-scan mode. In the first scan, topography data were obtained in the dynamic mode (also known as tapping mode). In the second scan, the tip was raised to 100 nm, and the surface potential was recorded by following the topography contour registered in the first scan. Topography and surface potential maps were collected ex-situ in an air atmosphere at 22 °C and a relative humidity of approximately 30%. A pixel resolution of 512 × 512, a zero- DC bias voltage, and a scan frequency rate of 0.3 Hz were used in all AFM/SKPFM measurements. Histogram analysis based on the multimodal Gaussian distributions was used to interpret the topography, amplitude, and surface potential distribution on the nodular surface microstructure.

#### XRD

The crystallinity of the TDC coating was evaluated using grazing incidence X-ray diffraction (GIXRD) analysis, while the carbon steel substrate was characterized separately through standard XRD analysis. The analysis utilized Bragg–Brentano focusing geometry with Co Kα radiation. A Bruker D8 Advance X-ray diffractometer, equipped with a graphite monochromator and a Vantec position-sensitive detector, was employed. The XRD parameters included a divergence slit Var20, a scatter screen height of 9 mm, and operating conditions of 40 kV and 40 mA. Data for TDC coating in GIXRD analysis were collected in coupled θ–2θ mode, over a scan range of 20° to 120°, with a step size of 0.021° and a counting time of 1 s per step. All XRD results were processed using Bruker software Diffrac.Suite.EVA vs 7.1, AbsorbX.

#### XPS

XPS was used in this study to determine the chemical state(s) of the oxide layer on the TDC coating before and after exposure to NaCl solution. XPS analyses were performed using a PHI Versaprobe II (Physical Electronics) spectrometer equipped with a monochromatic Al Kα X-ray source (1486.6 eV photon energy). The binding energy range was calibrated using the Cu 2p_3/2_ (932.62 ± 0.1 eV) and Au 4f_7/2_ (83.96 ± 0.1 eV) lines. Spectra were recorded at a takeoff angle of 45° with an irradiation power of 49.6 W, corresponding to a beam diameter of 200 μm. A 25 × 10 × 4 mm flat carbon AISI 52100 steel plate coated with TDC coating was used for the analysis. This sample was mounted onto the specimen holder using double-sided tape to insulate the sample from the ground. Measurements were performed using the built-in charge neutralizing system (which utilizes a combined low energy electrons and ion beam) and settings were calibrated with a PET reference sample (fwhm O=C–O C 1s peak <0.85 eV). Based on the absence of tails in the C 1s and O 1 s spectra the neutralizer settings used were sufficient to neutralize most differential charging effects in this coating, although some peak broadening due to differential charging could not completely be excluded. Survey scans recorded in the energy range 1400–0 eV at a pass energy of 187.5 eV, a step size of 1 eV, and a time per step of 50 ms (10 sweeps), revealed the presence of minor traces of N and Na next to Cr, O and C. High-resolution scans of the O 1s (30 sweeps), Cr 2p (90 sweeps) and the C 1s (30 sweeps) regions were recorded using a pass energy of 23 eV, a step size of 0.1 eV and a time per step of 50 ms. Chamber pressure during the measurements was ∼7.5 × 10^–9^ Torr due to the inflow of Ar ions in the chamber during neutralization. All spectra were charge-corrected relative to the adventitious carbon C 1s peak, set at 284.8 eV. Data analysis was carried out using PHI Multipack software (V9.9.2). The thickness of the passive film on various exposed surfaces was estimated using the Strohmeier equation, taking into account the predominant formation of Cr_2_O_3_ and Cr(OH)_3_:^23^
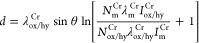
1All parameters of the equation can be described as follows: inelastic mean free paths of metallic Cr (λ_m_^Cr^ = 1.55 nm), Cr(OH)_3_ (λ_hy_^Cr^ = 2.27 nm), and Cr_2_O_3_ (λ_ox_^Cr^ = 1.83 nm), volume density of metal (*N*_m_^Cr^ = 7.19 g/cm^3^), Cr(OH)_3_ (*N*_hy_^Cr^ = 3.11 g/cm^3^), and Cr_2_O_3_ (*N*_ox_^Cr^ = 5.22 g/cm^3^), the peak fitted area percentages of the metallic Cr (*I*_m_), Cr(OH)_3_ (*I*_hy_^Cr^), and Cr_2_O_3_ (*I*_ox_^Cr^) signals, and the photoelectron takeoff angle (θ = 45°, corresponding to 0.785 radians). The inelastic mean free paths of metallic chromium, oxide, and hydroxide were extracted from the NIST Electron Inelastic-Mean-Free-Path Database (SRD 71).

#### AC/DC Multi-electrochemical Analyses

All electrochemical measurements were conducted using a conventional three-electrode electrochemical cell equipped with a Biologic SP 300 multichannel potentiostat. The setup included an Ag/AgCl/KCl3M reference electrode (+222 mV vs SHE), a platinum mesh as the counter electrode, and the coated samples as the working electrodes. The electrolyte used was a 3.5 wt % NaCl solution, prepared with ultrapure water (Milli-Q ix7003, >5 MΩ·cm) and NaCl salt (J.T.Baker), serving as an aggressive medium. The pH of the 3.5 wt % NaCl solution was measured using a pH meter at 22 ± 1 °C, with a value of 5.6 recorded during the experiment. Prior to all electrochemical measurements, the samples were immersed in the 3.5 wt % NaCl solution for 1 h to allow stabilization of the open-circuit potential (OCP) and to achieve a steady-state condition. Potentiodynamic polarization (PDP) tests were performed at a scan rate of 1 mV/s, starting from 100 mV below the OCP (cathodic) to 1000 mV vs Ag/AgCl (anodic). Electrochemical impedance spectroscopy (EIS) was conducted over a frequency range of 100 kHz to 10 mHz, using a sinusoidal excitation signal of ±10 mV. Additionally, the electronic properties of the passive film formed on the TDC coating were evaluated using Mott–Schottky (MS) analysis. Mott–Schottky analysis was performed using multifrequency EIS measurements.^12,24^ The TDC sample was polarized with a potential step of 50 mV, starting at −400 mV and extending to 1000 mV vs Ag/AgCl, following a 1h exposure in a 3.5 wt % NaCl solution. An amplitude of ±10 mV was applied during the measurements. Since the Bode magnitude exhibits a negative slope starting around 6 kHz (indicating the onset of capacitive reactance influencing the polarization response), 6 kHz was selected as the reference point for the MS plot. The MS analysis is based on the following equation:
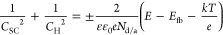
2where *C*_SC_ is the space charge capacitance, *C*_H_ is the capacitance of the Helmholtz double-layer, *E* is the applied potential, ε is the dielectric constant of the complex oxide film, ε_0_ is the vacuum permittivity (8.854 × 10^–14^ F·cm^–1^), *e* is the electron charge (1.6 × 10^–19^ C), *N*_d_ is the donor density, *N*_a_ is the acceptor density, *E*_fb_ is the flat band potential, and *k* and *T* are the Boltzmann constant and absolute temperature, respectively. All samples were cleaned in acetone for 2 min, using a low-level ultrasonication process.

## 3 Results and Discussion

### 3.1 Morphological, Microstructural, and Electronic Surface Characteristics of TDC Coatings

**Figure 1:**
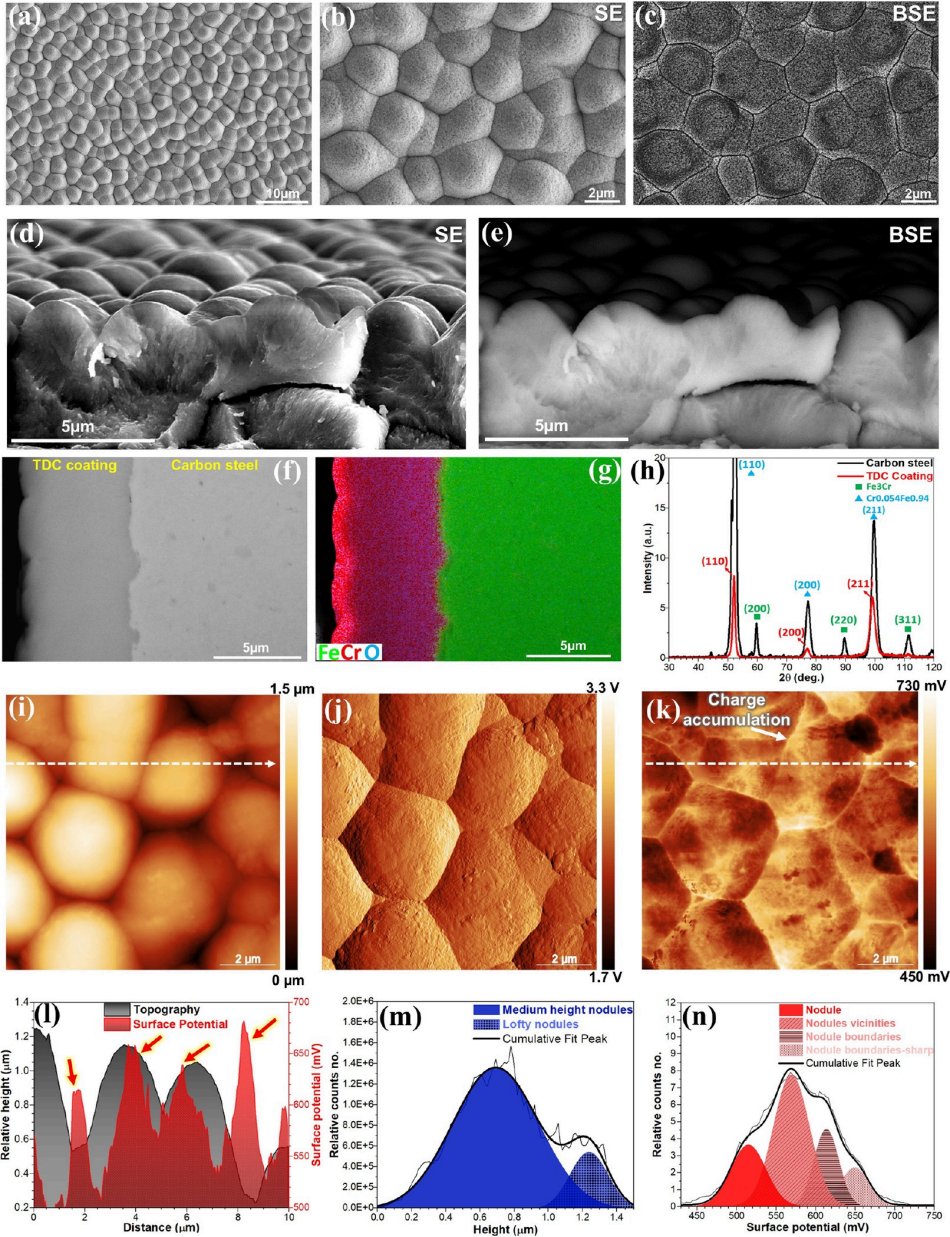
(a–c) Top-view SEM images of the TDC coating surface at low and high magnifications, capturing both secondary (SE) and backscattered electron (BSE) signals. (d, e) Cross-sectional SE and BSE-SEM images of the TDC coating, were prepared by fracturing the coating to reveal the cross-section. (f, g) BSE-SEM image and EDS cross-sectional elemental maps of the TDC coating applied on a carbon steel substrate. (h) GIXRD patterns of the TDC coating plus XRD pattern of carbon steel substrate. (i–k) Topography, amplitude, and electrical surface potential maps of the TDC coating. (l) Line profiles of topography and surface potential corresponding to images (i) and (k). (m) Topography histogram and (n) surface potential histogram, both related to images (i) and (k), respectively.

Top-view SE-SEM images of the TDC coating, captured at both low and high magnifications (Figure 1a,b) reveal the distribution of its distinctive hierarchical nodular morphology. The nodules exhibit an average diameter of 3.6 ± 0.5 μm, accounting for 95.9% of the total surface area. The remaining 4.1% is attributed to the nodule boundaries, with a boundary length per unit area of 0.61 ± 0.02 μm/μm^2^. The backscatter signal in Figure 1c illustrates the nodule distribution and its boundaries, revealing a uniform distribution with no observable surface defects, including pinholes or microcracks. Additionally, the scanning electron (SE) and backscattered electron (BSE) SEM side-view images of the TDC’s fracture surface, shown in Figure 1d,e, discernibly reveal a compact structure devoid of defects, such as microcracks, as well as multiple crystalline branches (see next section for further details). Besides, the SEM cross-section and the associated elemental maps from a polished surface (Figure 1f,g) noticeably display the TDC coating applied to the carbon steel substrate, demonstrating a coating thickness of 5 ± 0.5 μm, which varies with the base substrate surface roughness and morphology. An analysis of the microstructural and morphological characteristics reveals that conventional hard chromium coating significantly differs from TDC, demonstrating a uniform morphological distribution, as shown in Figure S1. AFM imaging and histogram analysis indicate that the hard chromium coating has a mean surface roughness of 12.7 ± 5 nm (Figure S1). However, a significant drawback is the presence of pronounced microcracks in the hard chromium layer (Figure S1). These microcracks are clearly visible in the SEM/EDXS cross-sectional images (Figure S1), showing that they extend into the substrate. This severely compromises the mechanical and corrosion resistance of the hard chromium coating, exacerbating galvanic corrosion and accelerating the anodic dissolution of the substrate (as discussed further below). Concerning the crystallographic texture of the TDC coating analyzed through the grazing-incidence X-ray diffraction (GIXRD) technique (Figure 1h), two major intensive diffraction peaks, Cr(110) and Cr(220) crystal planes, as well as a smaller peak corresponding to Cr(200) were detected (Library matching information in Figure S2). This provides additional evidence of the preferential orientation of the TDC crystalline structure along the Cr(110) and Cr(220) crystal planes.

To evaluate the surface physical and electrical characteristics of the TDC coating, topography, amplitude, and electrical surface potential/charge maps were generated, as shown in Figure 1i–k. The integrated local topography and amplitude mappings provide an evident view of the nodules on TDC coating surfaces, highlighting their slightly different heights and the distribution of nodule boundaries, with a root-mean-square (*R*_q_) of 241 ± 50 nm. The height histogram derived from the topography image in Figure 1m reveals two distinctive peaks, indicating the presence of nodules with mean heights of 0.69 μm for medium elevation and 1.23 μm for lofty elevation. A noteworthy observation is that the electrical surface potential and/or charge signals reveal a correlation between potential variations and the large nodules seen in the topography (Figure 1k). The highest surface potential and/or charge values are predominantly found near nodules, especially at their boundaries, as indicated by the red arrows in the line profiles presented in Figure 1l. It has been documented that localized charges at grain or nodule boundaries can lead to the creation of defect states, which in turn bend energy levels and establish a contact potential difference (ΔCPD) with a space charge region in nodular boundary areas.^25,26^ The surface potential histogram presented in Figure 1n reveals a heterogeneous distribution of surface potential and/or charge, as demonstrated by the multiple deconvoluted peaks. Nonetheless, the TDC coating displays a notable surface potential range of 400 to 700 mV vs PtIr, indicating a high level of surface nobility or high work function (being further supported by the SKPFM cross-section shown in Figure S3). This implies a diminished likelihood of electrochemical activity and reduced charge transfer/mass transport at the solid/electrolyte interface.^27^

### 3.2 Exploring the Near-Nanocrystalline Structure and Chromium Compact Bilayer Oxide

**Figure 2:**
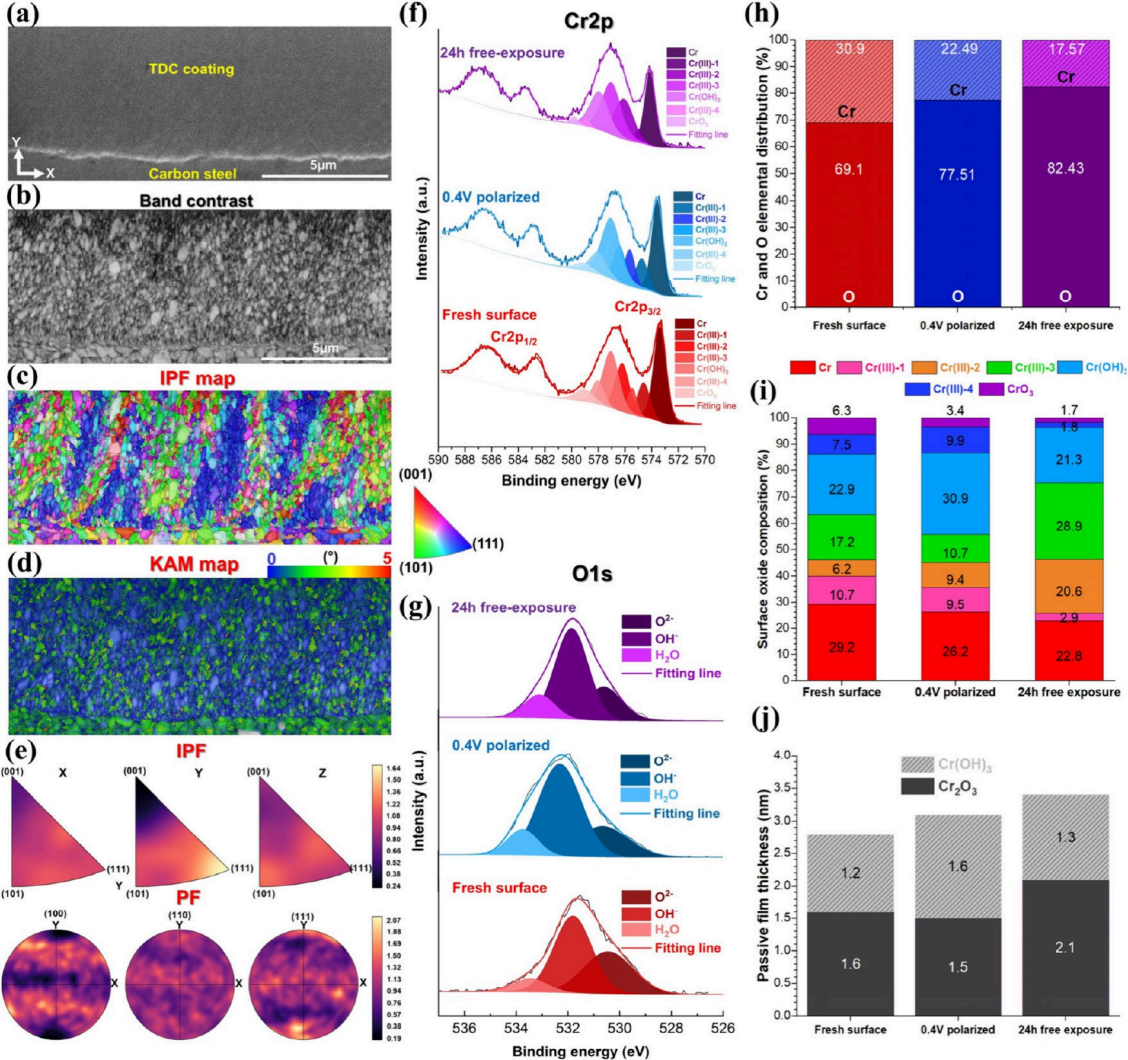
(a) Cross-sectional SEM images of mirror-polished TDC coating and (b–e) corresponding EBSD signals including (b) band contrast, (c) IPF color map, (d) KAM, and (e) IPF and pole figure. (f, g) XPS spectra of the (f) Cr 2p and (g) O 1s electron energy regions on the TDC coating surface were obtained under three different conditions: fresh surface, polarized surface at 400 mV vs Ag/AgCl in 3.5 wt % NaCl for 10 min, and after 24 h of free exposure to 3.5 wt % NaCl. (h–j) The analysis includes (h) the surface oxide composition, (i) the various oxidation states of chromium, and (j) the estimated thickness of the bilayer oxide (i.e., passive film) on the TDC surface derived from high-resolution XPS spectra of Cr 2p.

A detailed analysis of the crystalline structure, grain size, and orientation provides valuable insights into the physicochemical evolutions to be expected at the solid/electrolyte interface upon immersion in an electrolyte, with particular relevance to understanding corrosion mechanisms. Figure 2a–c presents the SEM and EBSD cross-sectional analysis of the TDC coating, featuring the associated band contrast and inverse pole figure (IPF) color map. The band contrast image distinctly reveals a near-nanocrystalline structure, with an average grain size of 228 ± 75 nm and 84.5% of grain boundary angles exceeding >10°. The TDC coating exhibits a structure where near-nanoscale grains aggregate into clusters, displaying polycrystalline characteristics (see Figure 2c). During electrodeposition, the aggregation of grains results in the formation of multicrystal branches as poly near-nanograins coalesce and reorganize, predominantly along the (111) crystalline plane^28^ (highlighted in blue in Figure 2c). This process also produces slightly higher strain gradients (illustrated in Figure 2d). The IPF and pole figure (Figure 2e) display a wide distribution of grain orientations in the TDC coating. Nonetheless, certain crystal branches predominantly aligned with the ⟨111⟩ growth direction. These crystal branches are observable from the SEM side-view images of the fracture surface in Figure 1d,e. Figure 2d, which presents the kernel average misorientation (KAM) map, highlights that grains within the ∼170–300 nm range display the highest KAM values. Evidence points to a high dislocation density, where crystal lattice curvature and local misorientations stem from an elevated concentration of geometrically necessary dislocations (GNDs).^29,30^ Overall, the near-nanocrystalline structure offers a heightened surface area-to-volume ratio due to its increased density of grain boundaries, which enhances electrochemical oxidation kinetics and drives the formation of stable passive films.^31^

The TDC coating forms a nanometer-scale bilayer surface oxide, which displays diverse oxidation states under various conditions, particularly in an aggressive environment (*vide infra*). In this study, these include a freshly prepared surface, polarization at 400 mV vs Ag/AgCl, and after 24 h of exposure to a 3.5% NaCl solution. Figure 2f shows high-resolution XPS spectra for Cr 2p and its associated O 1s signals (Figure 2g), corresponding to different surface conditions. The Cr 2p spectra were deconvoluted into multiple components, identifying metallic Cr^0^, Cr(OH)_3_, and several Cr(III) species, marked by the characteristic multiplet splitting of Cr^3+^.^32^ Interestingly, Cr(III) oxides display a well-defined multiplet structure, while Cr(OH)_3_ exhibits a broader, less distinct peak. Additionally, the single peak observed at a binding energy of 579.6 eV for all exposed surfaces can be attributed to the absence of unpaired electrons in Cr(VI) compounds, specifically CrO_3_.^33^ This aligns with the XRD results, which reveal three distinct peaks corresponding to CrO_3_ (Figure S2). Table S1 provides the binding energy details for all the Cr 2p fitted peaks. Comparing the Cr 2p spectra of the TDC coating across three different exposed surfaces discloses that the sample polarized at 400 mV vs Ag/AgCl in 3.5% NaCl demonstrates increased Cr(OH)_3_ formation and a reduction in Cr_2_O_3_ content. However, over 24h of exposure, the amount of Cr(OH)_3_ hydroxide decreases while Cr_2_O_3_ oxides continue to grow (Figure 2f,i). The distribution analysis of O and Cr elements on the TDC surface oxide from the fresh surface to 24h of NaCl exposure shows an increase in O and a decrease in Cr signals, indicating more active surface oxidation and growth of the passive layer (Figure 2h). Overall, the O 1s spectrum displayed three prominent peaks corresponding to lattice oxide (O^2–^), hydroxide groups (OH^–^), and adsorbed water molecules, with binding energies of 530.4, 531.8, and 533.4 eV, respectively. XPS analysis reveals that the passive film consists of a bilayer structure, with Cr(OH)_3_ as the outer layer and Cr_2_O_3_ as the inner layer (Figure 2j). Initially, the native oxide film of the fresh surface reveals a slightly greater thickness for Cr_2_O_3_ (1.6 nm) compared to Cr(OH)_3_ (1.2 nm). After 24 h of exposure to a 3.5% NaCl electrolyte, the thickness of the oxide film formed by water significantly increases, reaching 2.1 nm for Cr_2_O_3_ and 1.3 nm for Cr(OH)_3_. The intricate bilayer oxide film, formed in water and native to the material, serves as a highly effective protective nanolayer.^34^ Its remarkably low degradation rate noticeably impedes charge transfer and mass transport at the Cr/oxide/electrolyte interfaces.^35^

### 3.3 Evaluating Electrochemical Activity and Corrosion Performance of TDC Coating

**Figure 3:**
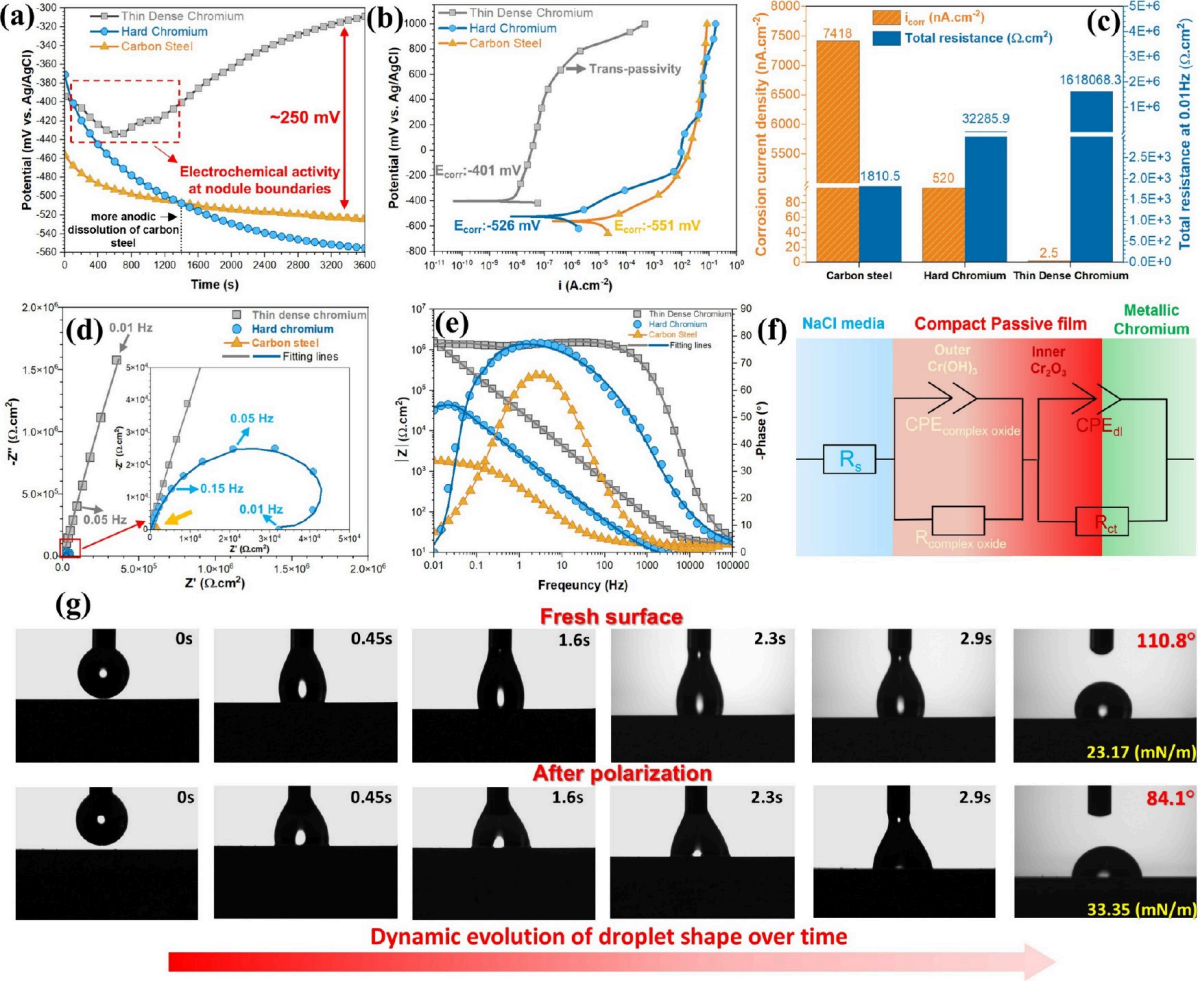
(a) OCP curves. (b) PDP curves of TDC, hard chromium coatings, and carbon steel substrate during exposure to 3.5 wt % NaCl solution. (c) Corrosion current density (*i*_corr_) and total resistance at 0.01 Hz for TDC, hard chromium coatings, and carbon steel, obtained from (b) and (d), respectively. (d) Nyquist plots and (e) Bode phase and amplitude plots for both coatings and carbon steel after 1 h of OCP monitoring in 3.5 wt % NaCl solution. (f) Electrical equivalent circuit (EEC) model used for accurate fitting of the TDC EIS curve. (g) Dynamic shape evolution of a saline single droplet on a fresh TDC surface and after PDP testing, showing variations in contact angle and surface tension.

The electrochemical activity, bilayer oxide characteristics, and corrosion behavior of TDC and hard chromium coatings were evaluated through short-term exposure in a 3.5 wt % NaCl electrolyte, using open circuit potential (OCP), potentiodynamic polarization (PDP), and electrochemical impedance spectroscopy (EIS) analysis. The OCP curve of the TDC coating in Figure 3a exhibits an initial decrease in corrosion potential from −390 to −435 mV vs Ag/AgCl. Following minor fluctuations in the range of 690–1120s, the potential gradually rises, attributed to the formation and growth of a complex oxide layer.^36^ The hard chromium coating exhibited an initial corrosion potential of −370 mV vs Ag/AgCl, which closely matched that of the TDC coating. However, due to the presence of microcracks in its microstructure, the corrosion potential sharply declined, eventually dropping below that of carbon steel after approximately 1400 s. This behavior can be explained by the relatively strong galvanic coupling between chromium, a noble metal, and the highly electrochemically active steel substrate, which accelerates the anodic dissolution of the steel.

The corrosion current density (*i*_corr_) and potential (*E*_corr_) for both carbon steel and hard chromium were determined using Tafel extrapolation, incorporating the intersection of tangents to cathodic and anodic branches. However, for the TDC coating, the cathodic branch was either poorly defined or unsuitable for accurate fitting due to passivation effects. Consequently, the *i*_corr_ was determined as the intersection of the tangent to the anodic branch and the line through the *E*_corr_.^37,38^ Upon comparing the PDP curves of TDC and hard chromium coatings immediately after the EIS measurement in Figure 3b, it is evident that the TDC coating experiences a shift of nearly 2 orders of amplitude in corrosion current density. This results in a remarkably low corrosion current density (*i*_corr_ = 2.5 nA·cm^–2^), significantly outperforming hard chromium, which exhibits a much higher (*i*_corr_ = 520 nA·cm^–2^) current density. Furthermore, after a short activation region at −365 mV vs Ag/AgCl, the TDC coating entered a passivation region, characterized by an extended and stable passive range. The overpotential shifted toward more positive values, reaching up to 1000 mV vs Ag/AgCl, with no visible indications of pitting corrosion (Figure S4). SEM and BSE-SEM images of the TDC coating following the polarization test at a high voltage of 1000 mV vs Ag/AgCl revealed only a slight, infrequent localized corrosion (trenching process) along the nodule boundaries. This gradual deterioration is attributed to the high-energy sites at these boundaries, which enhance electrochemical activity (Figure S5).^39^ Besides, this phenomenon can also be linked to transpassive behavior, resulting from the progressive degradation of the compact protective oxide layer. These findings correlate with the electrical surface potential results obtained by SKPFM, indicating greater surface potential and/or charge accumulation at these nodule boundaries (Figure 1k).

The PDP curve of hard chromium exhibits a striking similarity to those of carbon steel, characterized by an extensive range of activation regions, particularly in terms of corrosion potential (*E*_corr/hard chromium_ = −526 mV and *E*_corr/carbon steel_ = −551 mV vs Ag/AgCl). The key observation is the similar electrochemical signals and corrosion performance exhibited by both carbon steel and hard chromium at overpotential values of −165.4 mV vs Ag/AgCl. This similarity strongly suggests that aggressive electrolytes diffuse through the microcracks, ultimately reaching the carbon steel substrate and completely damaging the coating (Figures S6 and S7). The macrograph images in Figure S7 clearly illustrate a contaminated electrolyte, showing significant iron release and corrosion products from the carbon steel beneath the hard chromium layer following PDP. The corrosion current density (*i*_corr_) results extracted from all PDP experiments are presented in Figure 3c. The findings demonstrate a remarkably low *i*_corr_ value of 2.5 nA·cm^–2^ for the TDC coating, achieving a corrosion resistance that is nearly ten times lower than that of hard chromium (520 nA·cm^–2^). The superior performance highlights the coating’s effectiveness in suppressing redox reactions, charge transfer, and mass transport at the Cr/oxide/electrolyte interface.

Figure 3g illustrates the dynamic changes in NaCl droplet shape on the TDC coating surface before and after PDP at 1000 mV vs Ag/AgCl. On the fresh surface, the NaCl droplet exhibits limited wettability, with a contact angle (CA) of 110.8°, a total surface tension (γ^total^) of 23.17 mN·m^–1^, and a hydrophobicity behavior. After polarization, however, the TDC surface shows a marked increase in droplet wettability, as indicated by a lower CA (84.1°) and higher γ^total^ (33.35 mN·m^–1^), indicating a transition to a hydrophilic surface. This alteration in surface behavior is ascribed to the intricate physicochemical evolution of the micronodular structure and its nanoscale characteristics, following the Cassie–Baxter theory.^40,41^ Nevertheless, detailed analyses via AFM/SKPFM, XPS, and SEM indicate that increased anodic dissolution and Cr oxidation, a greater predominance of Cr(OH)_3_ over Cr_2_O_3_, combined with changes in surface roughness and electronic potential, together modulate the surface wettability and interaction with droplets.

An analysis of the EIS data for both TDC and hard chromium coatings, as shown in Figure 3d,e, reveals notable differences. When comparing the Nyquist plots for all samples, the TDC coating exhibits significantly higher real (*Z*′) and imaginary (−*Z*″) impedance. This indicates that the TDC possesses the highest charge transfer resistance (polarization resistance) and demonstrates superior corrosion resistance.^12^ In contrast, the hard chromium coating displays a small capacitive loop with low impedance, accompanied by an inductive loop at low frequencies in the Nyquist diagram. This indicates potential microcracks and localized corrosion processes, resulting in a remarkably higher charge transfer at the solid/electrolyte interface.^11^ Utilizing the most applicable equivalent electrical circuit (EEC), the Bode phase diagram of TDC coating highlights two time-constant elements. The EEC model consists of solution resistance (*R*_s_), complex oxide (inner and outer oxides) resistance (*R*_complex oxide_), charge transfer resistance (*R*_ct_), constant phase element of complex oxide (CPE_complex oxide_), and double layer (CPE_dl_). Owing to defects, rough surface features, and material heterogeneities, the capacitance behaves imperfectly and is represented as a constant phase element (CPE) instead of a perfect capacitor.^42^ The CPE can be explained by the following equation:
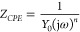
3where *Y*_0_ is the admittance of CPE, j is the imaginary unit, ω is the angular frequency, and *n* is the CPE exponent (−1 ≤ *n* ≤ 1, where −1, 0, and 1 are assigned to a system which is a pure inductor, pure resistance, and pure capacitance, respectively). The results of the fitted parameters are presented in Table S1. The Bode phase and amplitude diagrams of the TDC coating show that the capacitance onset occurs at slightly higher frequencies compared to both hard chromium and, in particular, carbon steel. This can be attributed to the complex and compact bilayer oxide, which exhibits significant electronic properties (as a very low charge transfer semiconductor) and electrochemical behavior at the Cr near-nanocrystalline interface, in contrast to the carbon steel substrate. Consequently, this results in a delayed capacitive response,^43^ which becomes evident at higher frequencies compared to carbon steel. Due to the exceptional properties outlined above, a high phase angle (∼80°) and a continuous, smooth distribution of phase angles are observed for TDC coating in the frequency range of 100 to 0.01 Hz. This behavior highlights the significant role played by the complex and compact bilayer oxide with a pseudodielectric behavior.^44^

According to prior studies,^45,46^ along with the presence of a well-defined and compact bilayer oxide on the TDC surface, consisting of a Cr(OH)_3_/electrolyte interface and a complex multioxide structure (indicated by the multiplet pattern of Cr(III) detected via XPS) at the Cr_2_O_3_/metallic Cr interface, we considered two-time constants in the series model, as illustrated schematically in Figure 3f. The double layer capacitance (*C*_dl_) of TDC coating shows a lower value (*C*_dl_ = 15.2 μF·cm^–2^) compared to the capacitance of complex oxide (*C*_complex oxide_= 52.1 μF·cm^–2^), suggesting greater charge transfer resistance at the bilayer oxide/metallic Cr interface. This is consistent with the significantly higher charge transfer resistance (*R*_ct_) value of 5.3 MΩ·cm^2^ at the bilayer oxide/metallic Cr interface, as compared to the *R*_complex oxide_, which is 69.5 kΩ·cm^2^.^46^

### 3.4 Electronic Properties and Long-Term Durability of TDC Complex Bilayer Oxide

**Figure 4:**
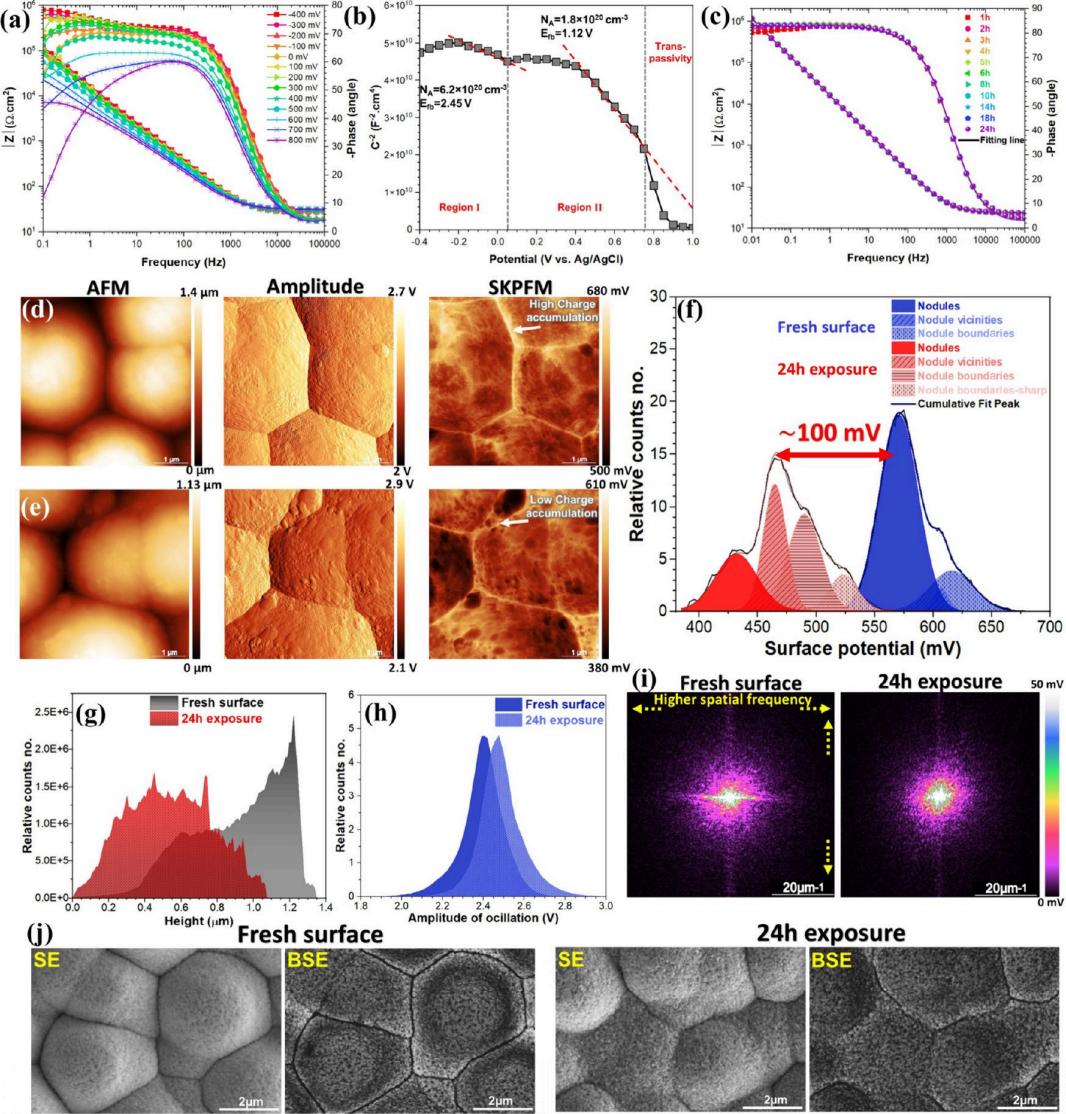
(a) Dynamic EIS analysis (Bode phase and amplitude plots) of the TDC coating surface from −400 mV to 800 mV vs Ag/AgCl after 1 h of exposure to 3.5 wt % NaCl solution. (b) Mott–Schottky plot of the TDC coating surface after 1 h of exposure to 3.5 wt % NaCl solution. (c) Monitoring of Bode phase and amplitude plots for the TDC coating during 24 h of exposure in 3.5 wt % NaCl solution. (d, e) Topography, amplitude, and electrical surface potential maps of the TDC coating in (d) fresh surface and (e) after 24 h exposure to 3.5 wt % NaCl solution. (f) Surface potential and (g) topography histograms related to images (d) and (e). (h) Amplitude histogram related to the cantilever oscillation on various TDC surfaces. (i) 2D-FFT surface potential maps. (j) Top-view SEM images of the TDC coating surface in two different conditions, capturing both secondary (SE) and backscattered electron (BSE) signals.

Dynamic-potential electrochemical impedance spectroscopy (EIS) was exploited to probe the evolving impedance and semiconductor behavior of the complex bilayer oxide on the TDC surface, as well as its acceptor density (e.g., p-type) in a 3.5 wt % NaCl solution (Figure 4a). The corresponding Mott–Schottky curve is presented in Figure 4b. Figure 4a illustrates that with increasing overpotential, leading to enhanced anodic dissolution and/or oxidation reactions on the TDC surface, the Bode diagrams for both amplitude and phase shift downward (mainly in the frequency range of 1000–0.1 Hz), indicating a decrease in total resistance and phase angle. Additionally, the presence of two dominant time constants in the diagrams becomes progressively less distinct due to the greater release of Cr metal ions and the enhanced formation of Cr(OH)_3_ hydroxide compared to Cr_2_O_3_, as revealed by high-resolution XPS results of Cr 2p at 400 mV vs Ag/AgCl in Figure 2f. The Mott–Schottky curve in Figure 4b highlights three distinct regions. Region I is defined by a low C^–2^ value (i.e., space charge capacitance). Region II exhibits a higher C^–2^, indicative of increased oxide/hydroxide film formation. In the trans-passivity region, the curve shows the significant dissolution of metallic Cr, pointing to chemical instability in the passive film and a gradual degradation process.^35^ Both regions I and II display negative slopes, which reflect typical p-type semiconductor properties indicating that Cr^3+^ vacancies are predominant point defects within oxide film.^47,48^ The differences in the p-type charge carrier density and flat band potentials in these regions are attributed to surface states, the inhomogeneous distribution of charge carrier density, and the dependency of carrier type and density on the sweeping potential.^49^ The passive film polarized between 150 mV and 750 mV vs Ag/AgCl displays a lower p-type charge carrier density (*N*_a_ = 1.8 × 10^20^ cm^–3^) and flat band potential (*E*_flat_ = 1120 mV vs Ag/AgCl) relative to Region I (*N*_a_ = 6.2 × 10^20^ cm^–3^, *E*_flat_ = 2450 mV vs Ag/AgCl), implying fewer charge carriers for electrochemical processes and greater chemical stability of the passive layer.^35^ To deepen the understanding of the electrochemical activities and corrosion resistance of the TDC coating, particularly its protective complex oxide film, extended EIS monitoring was utilized, as presented in Figure 4c. Except for the first 1h of exposure in 3.5 wt % NaCl, all Bode phase and amplitude plots exhibit a similar and highly stable impedance over the 24h period. This further confirms the exceptional physicochemical stability of the complex bilayer oxide and the TDC near-nanocrystalline structure against the degradation process.

From the topographic images (Figure 4d,e) of TDC coatings taken before and after 24h of exposure to 3.5 wt % NaCl, no noticeable variances in surface roughness, especially at the nanometric scale related to nodule height are observed. The AFM amplitude signal indicates the development of finer surface features and/or enhanced passivation across the nodules, shifting the amplitude histogram toward higher values (Figure 4h). The observed shift is consistent with the XPS findings (Figure 2) and is additionally confirmed by the SE and BSE-SEM images (Figure 4j). The SKPFM maps (Figure 4d,e) further reveal variations in the electrical surface potential, indicating an overall reduction in potential distribution by approximately Δ*E* = 100 mV (Figure 4f). This decrease is more pronounced at the nodule boundaries, which exhibit lower potential/charge accumulation, likely due to the enhanced formation of bilayer oxides (Cr(OH)_3_/Cr_2_O_3_).^50^ The water-formed Cr(OH)_3_/Cr_2_O_3_ oxides, functioning as semiconductors, regulate both the charge transfer and the surface potential while diminishing the electrostatic force interaction between the tip apex and the TDC surface.^51^ The 2D power spectral density (PSD) analysis in Figure 4i highlights a lower and mildly inhomogeneous surface potential distribution on the TDC surface after 24h of exposure, with a noticeable concentration at lower spatial frequencies.

**Figure 5:**
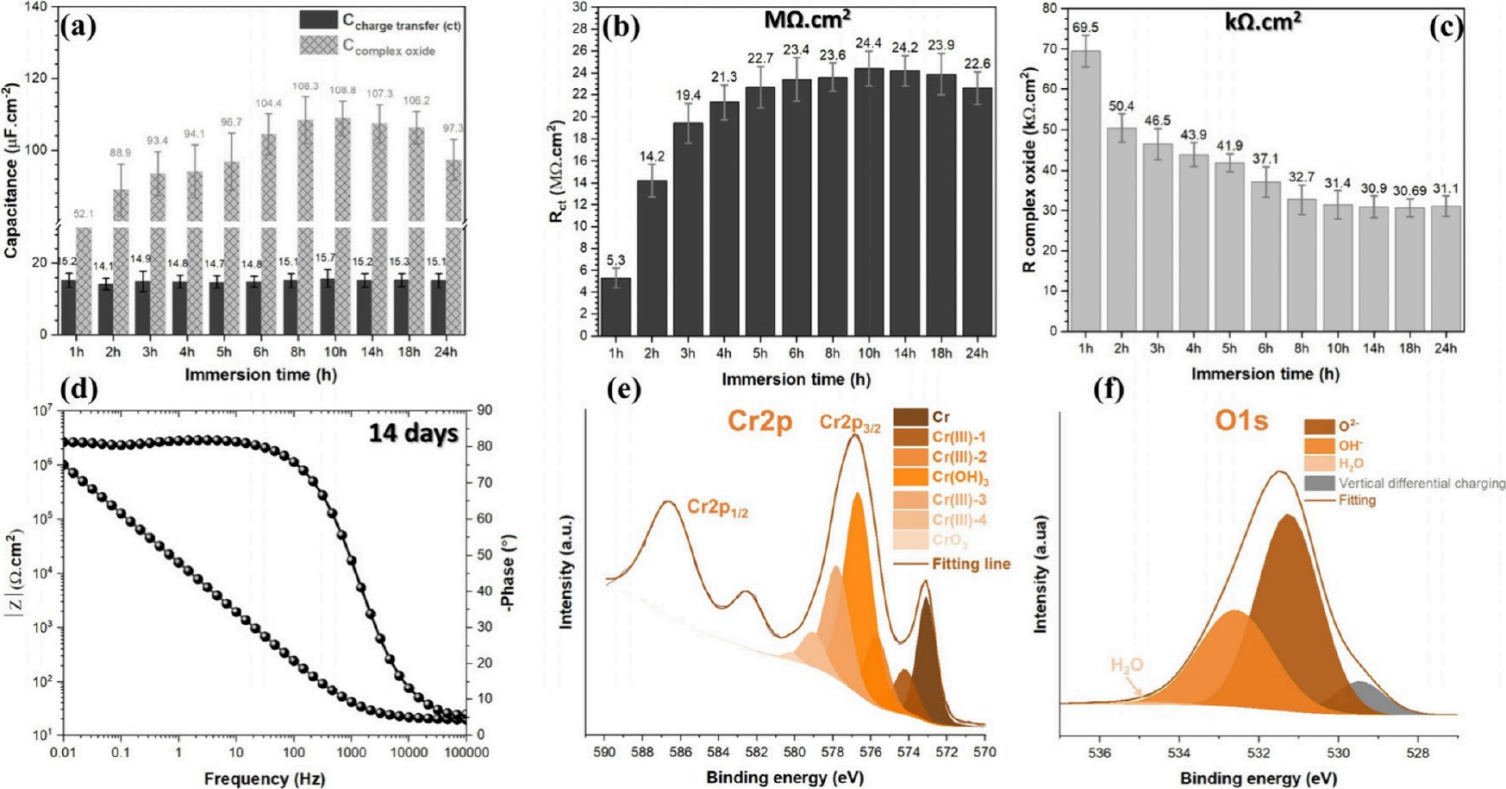
(a) Charge transfer and complex oxide capacitances, (b) charge transfer resistance (*R*_ct_), and (c) complex oxide resistance (*R*_complex oxide_) of the TDC coating surface, extracted from (c) and Table S2. (d) Bode phase and amplitude plot of TDC coating after 14 days exposure in 3.5 wt % NaCl solution. (e, f) XPS spectra of the Cr 2p and O 1s electron energy regions on the TDC coating surface after 14 days exposure in 3.5 wt % NaCl solution.

Figure 5a–c illustrate the capacitance and resistivity values for the complex bilayer oxide (Cr(OH)_3_ and Cr_2_O_3_)/electrolyte interface, referred to as *C*_complex oxide_, as well as the complex bilayer oxide/metallic Cr interface, labeled *C*_charge transfer(ct)_. These values are obtained from fitting analysis and detailed in Table S2. When comparing the values of *C*_complex oxide_ and *C*_ct_, it is evident that *C*_ct_ maintains stable behavior at low levels, while *C*_complex oxide_ increases steadily until the 8 h mark, after which it stabilizes. Additionally, the resistivity associated with these capacitances reveals a high *R*_ct_ value (MΩ·cm^2^), which shows a consistent upward trend throughout the exposure time. In contrast, *R*_complex oxide_ remains relatively low (in the kΩ·cm^2^ range) and follows a decreasing trend. By integrating the XPS results from fresh and 24 h exposed samples alongside the EIS parameters (Figure 2j), we observe that both Cr(OH)_3_ and Cr_2_O_3_ increase with exposure duration. Cr(OH)_3_ emerges as the more prominent outer layer of the bilayer oxide. This growth significantly impacts the capacitance increase and the notable decline in resistivity over time, marked by low bandgap energy that facilitates charge and mass transfer while also reducing chemical stability.^52^ Following 14 days of EIS monitoring, the TDC coating maintains stable Bode phase and amplitude responses, achieving a resistivity of around 1 MΩ·cm^2^ with no indications of localized corrosion initiation, as indicated in the SEM image in Figure S8. By comparison, the hard chromium coating exhibited total failure after only a single day of exposure (Figure S9). The thickness of bilayer oxide on the surface of TDC coating after 14 days of exposure reaches 1.6 nm for Cr(OH)_3_ and 2.5 nm for Cr_2_O_3_ (Figure 5e,f).

**Figure 6:**
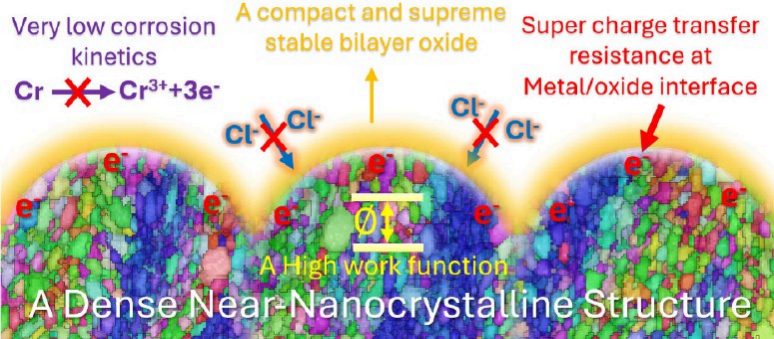
Schematic representation highlighting the exceptional physicochemical properties of the TDC coating and its superior corrosion resistance performance.

Finally, we compiled our findings and presented a schematic representation (Figure 6) to highlight how the near-nanocrystalline structure of thin, dense chromium coatings imparts unique physical and chemical properties, driving their remarkable corrosion resistance. The exceptional polarization resistance of the TDC coating is attributed to its near-nanocrystalline structure, characterized by a high density of grain boundaries and a substantial work function value (high nobility). These grain boundaries serve as diffusion pathways, facilitating the rapid formation of a stable, dense, and intricate nanometer-scale bilayer oxide. This reduces corrosion kinetics and enhances the coating’s protective performance.^53^ Furthermore, the near-nanocrystalline structures interfacing with nanometer-scale bilayer oxides can generate extra energy barriers, obstructing charge transfer and causing charge trapping.^51^ The dense near-nanocrystalline structure, combined with the rapid repassivation process, creates a formidable barrier that restricts chloride ion diffusion into the complex bilayer oxide, preventing their penetration and the breakdown of the passive film.

## 4 Conclusions

The nodular thin dense chromium (TDC) coating exhibits outstanding corrosion resistance, largely due to its compact, near-nanocrystalline structure and the formation of protective bilayer oxide. While electrical surface potential irregularities due to the hierarchical micronodular structure may create localized vulnerabilities, the overall TDC coating demonstrates exceptional chemical stability and passivation. Its dense near-nanocrystalline structure prevents defects like micro or nanocracks and inhibits the penetration of corrosive ions, contributing to its superior robustness. The coating’s protective bilayer oxide enhances corrosion resistance by reducing electrochemical activity and preventing charge transfer and mass transport, further strengthening its long-term corrosion performance. Over time, the bilayer oxide becomes thicker and more stable, boosting the TDC coating’s ability to resist corrosive attack. Overall, the TDC coating provides highly effective protection, making it a suitable candidate for highly challenging corrosion- and wear-resistant applications exposed to harsh environments.

## Supporting Information Available

Top view and cross-sectional SEM images of hard chromium; AFM and topography histogram of hard chromium; XRD patterns of carbon steel and GIXRD patterns of the TDC coating; AFM and SKPFM maps of the cross-sectional view of both the TDC coating and the carbon steel substrate; optical macrograph images of carbon steel, hard chromium, and TDC coatings after potentiodynamic polarization; SEM top-view images of the TDC coating after potentiodynamic polarization; SEM top-view images of hard chromium after potentiodynamic polarization; optical macrograph images of hard chromium after potentiodynamic polarization; SEM top-view images of the TDC coating after 14 days in 3.5 wt % NaCl solution; optical macrograph images of hard chromium and TDC coatings during EIS monitoring (PDF)
