# Supplementary material for: Advanced Nodular Thin Dense Chromium Coating: Superior Corrosion Resistance
Source: ACS Appl Mater Interfaces. 2025 Jan 22;17(5):8588–600. doi: 10.1021/acsami.4c19897 (PMC11803559; doi:10.1021/acsami.4c19897)
Supplement: Supplementary file 1 — am4c19897_si_001.pdf [file am4c19897_si_001.pdf]

Supporting Information

Advanced Nodular Thin-Dense Chromium Coating: Superior Corrosion Resistance

Ehsan Rahimi[a,b*] Thijs Nijdam[b], Adwait Jahagirdar[b], Esteban Broitman[b], Arjan Mol[a]

[a]Delft University of Technology, Department of Materials Science and Engineering, Mekelweg 2, 2628 CD Delft, The Netherlands

[b] SKF Research & Technology Development, 3992AE Houten, the Netherlands

E.Rahimi: **e.rahimi-2@tudelft.nl**

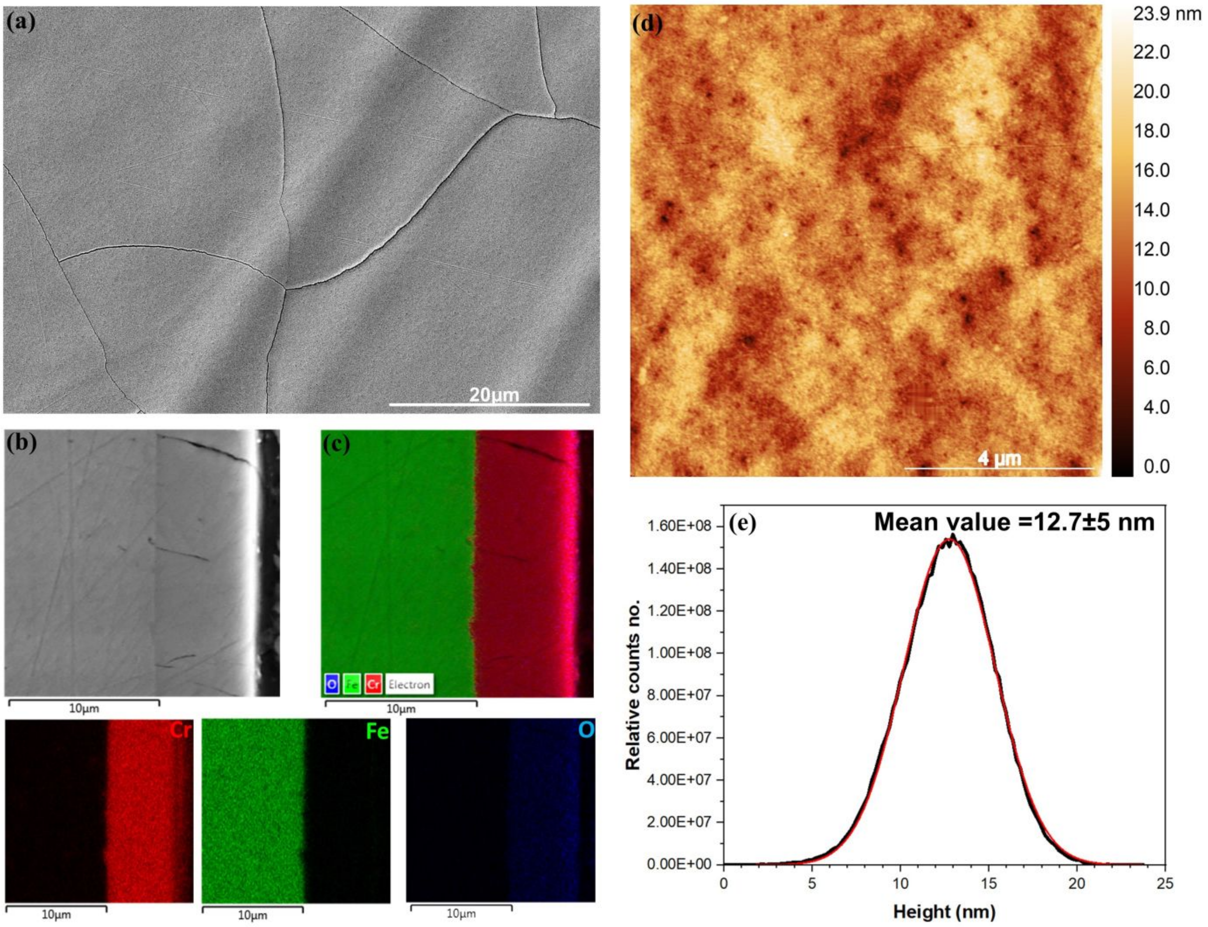

Figure S1. (a) Top-view SEM image of the hard chromium coating, highlighting the distribution of micro-cracks; (b) cross-sectional SEM image of the coating; and (c) corresponding EDS elemental maps showing the elemental distribution on the polished surface of the hard chromium; (d) topography map of hard chromium surface and (e) topography histogram obtained from (d).

.

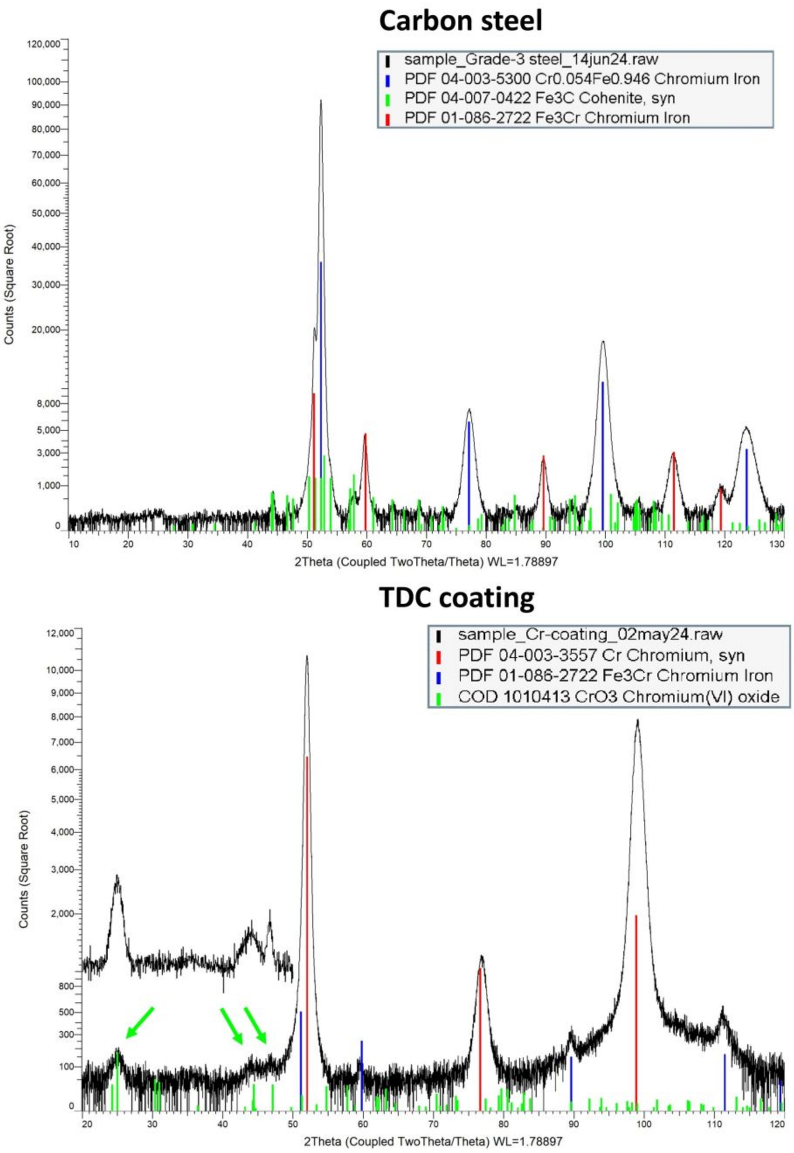

Figure S2. XRD patterns of carbon steel and GIXRD patterns of the TDC coating, matched with the corresponding XRD library.

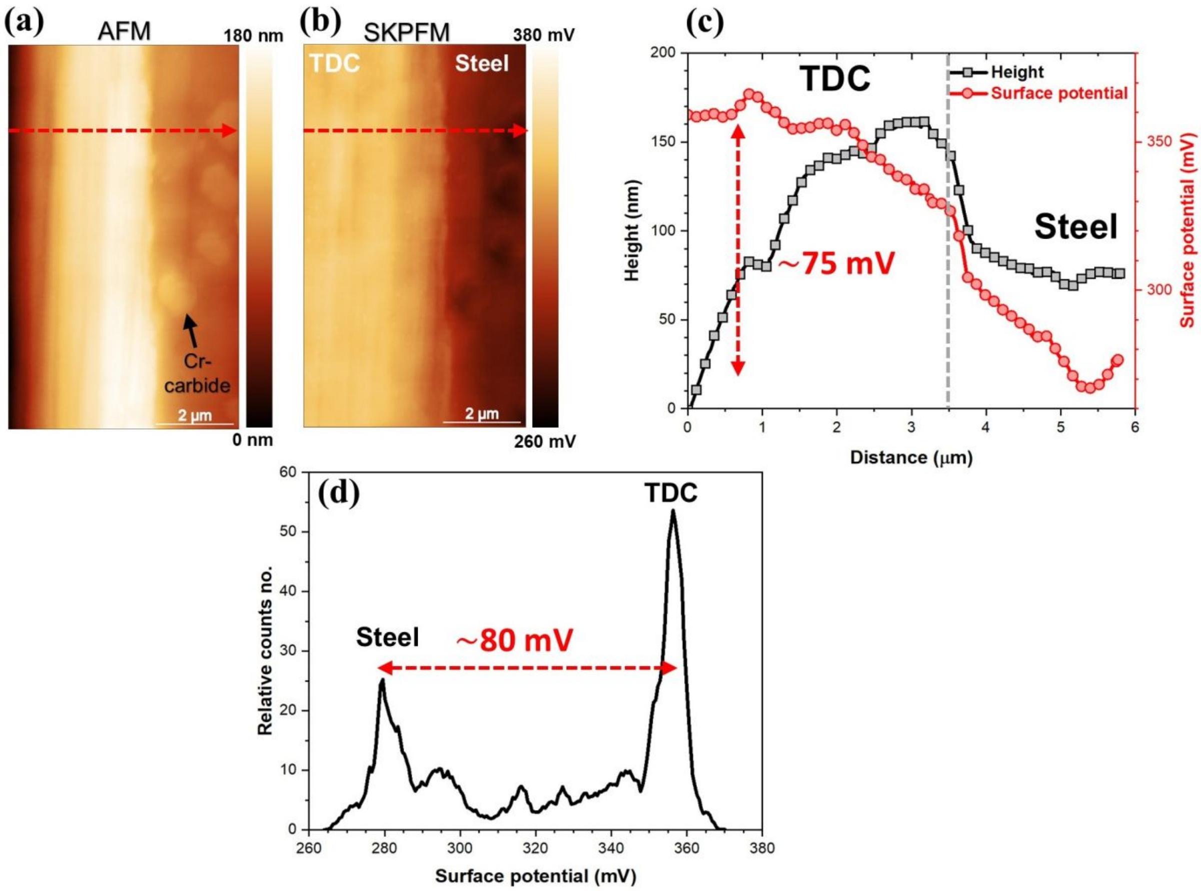

Figure S3. (a) AFM and (b) SKPFM maps of the cross-sectional view of both the TDC coating and the carbon steel substrate, (c) topography and surface potential line profiles corresponding to (a) and (b); and (d) histogram of the surface potential distribution extracted from (b).

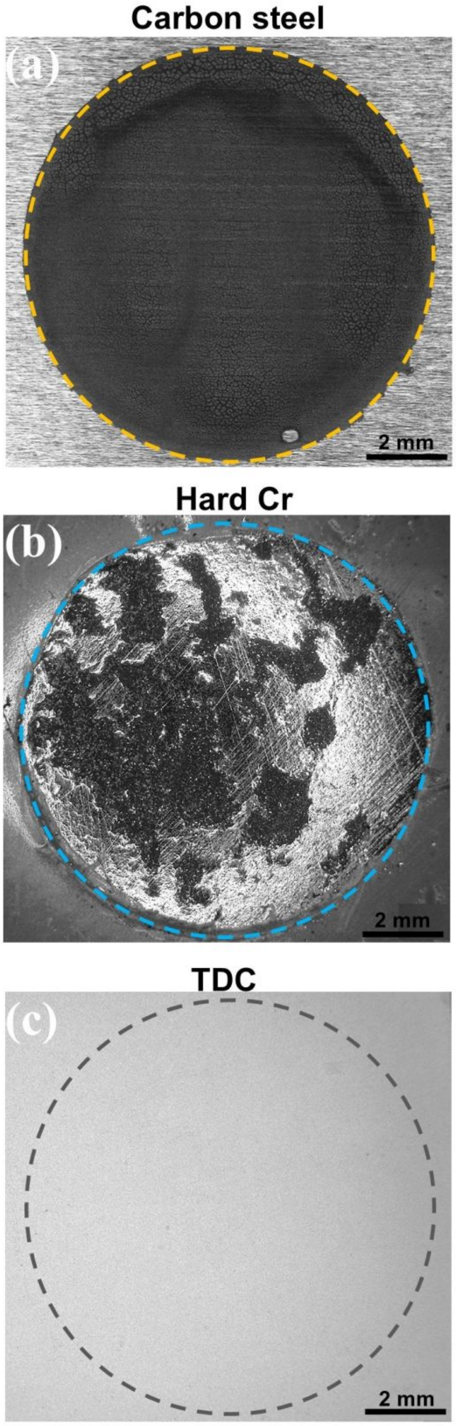

Figure S4. Optical macrograph images of (a) carbon steel, (b) hard chromium, and (c) TDC coatings after potentiodynamic polarization up to 1V vs. Ag/AgCl reveal significant differences in corrosion behaviour. Carbon steel was fully corroded, forming a black corrosion product, while hard chromium showed extensive delamination and coating detachment, attributed to the large accumulation of corrosion products.

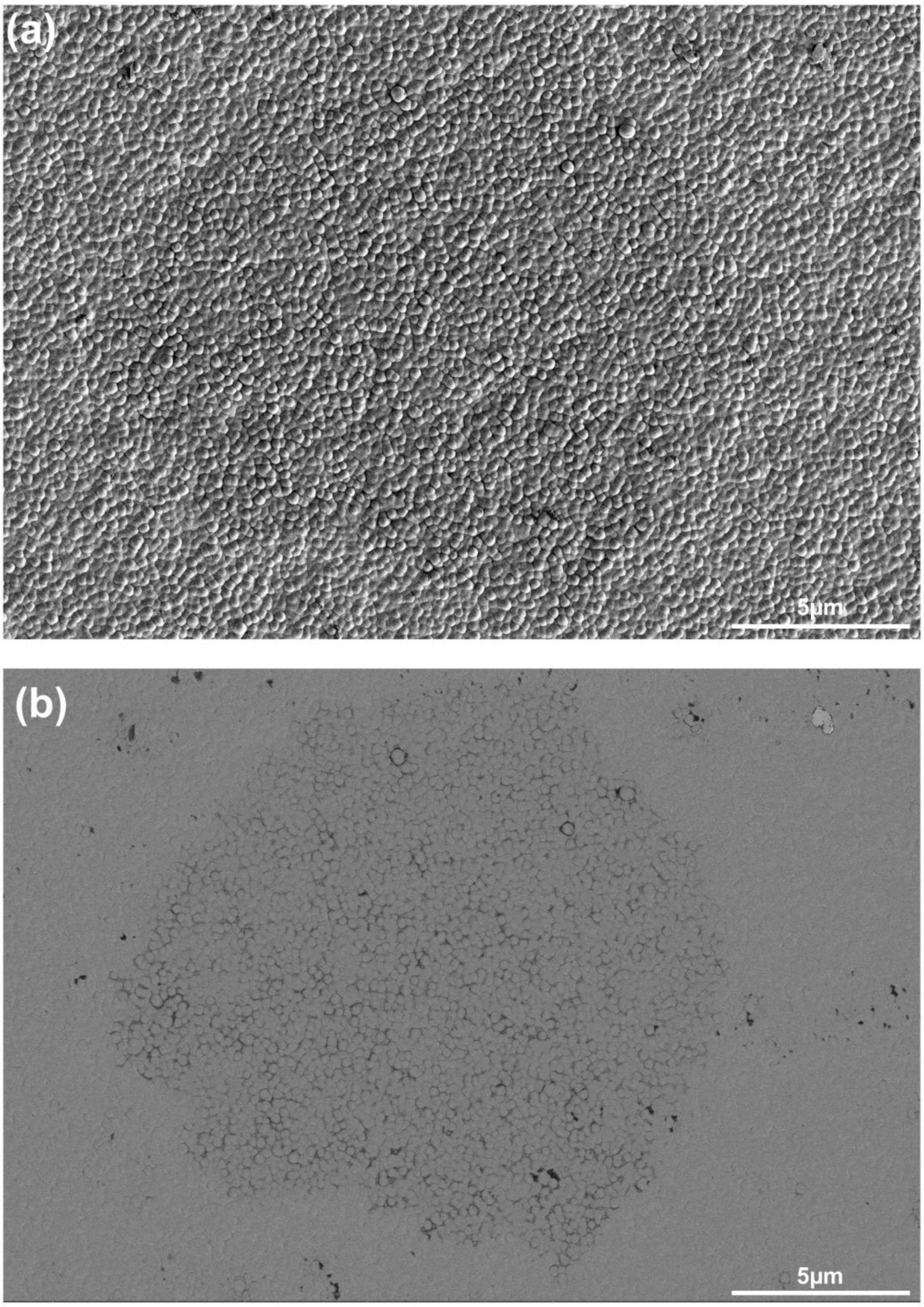

Figure S5. (a) SE and (b) BSE-SEM top-view images of the TDC coating after potentiodynamic polarization up to 1V vs. Ag/AgCl show no evidence of pitting or localized corrosion.

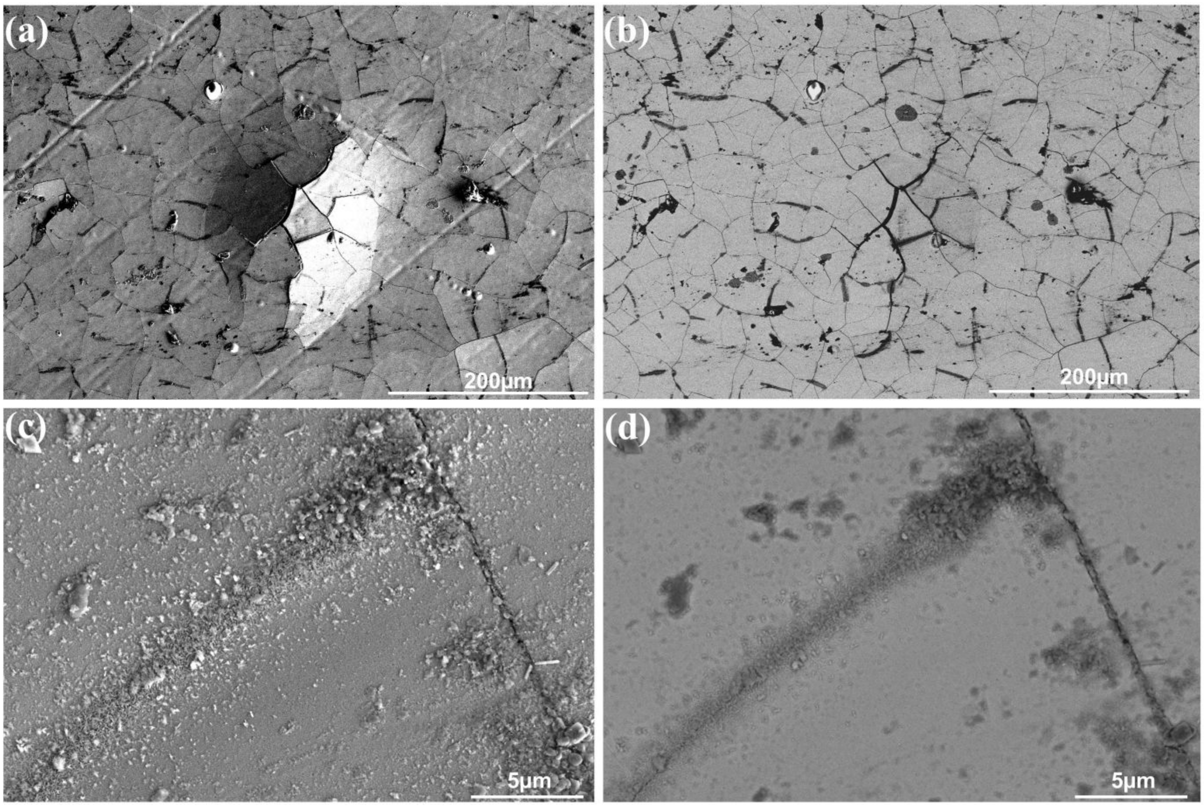

Figure S6. (a) SE and (b) BSE-SEM top-view images of hard chromium after potentiodynamic polarization up to 1V vs. Ag/AgCl reveal delamination of the coating, primarily due to the extensive formation of iron corrosion products. (c) SE and (d) BSE-SEM high-magnification images show corrosion products emerging from cracks in the coating.

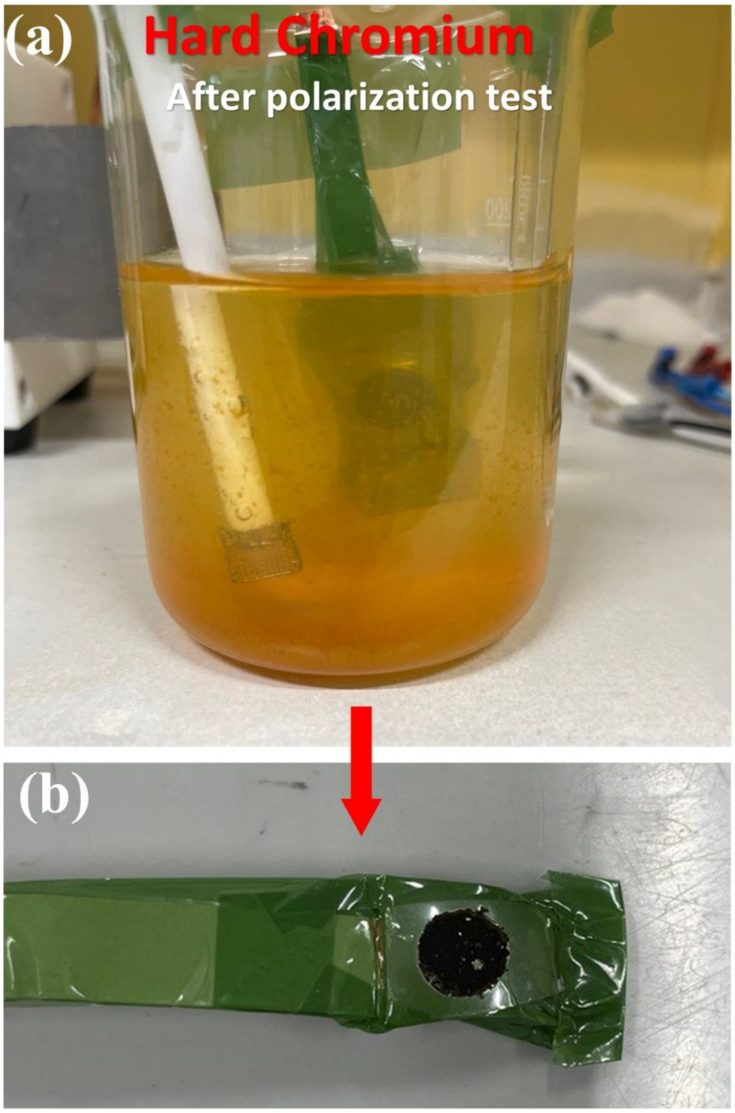

Figure S7. (a,b) Optical macrograph images of hard chromium after potentiodynamic polarization up to 1V vs. Ag/AgCl clearly show significant Fe ion release, deposition, and contamination of the solution.

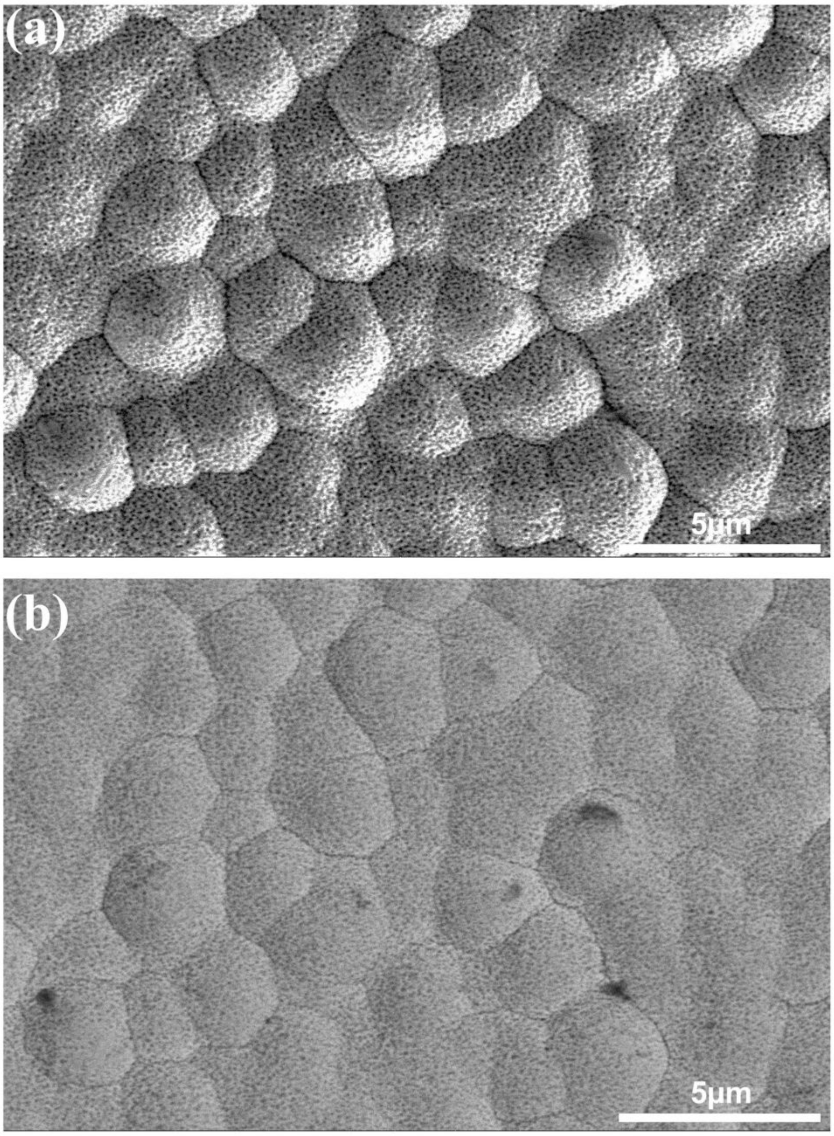

Figure S8. (a) SE and (b) BSE-SEM top-view images of the TDC coating 14 days in 3.5%wt. NaCl solution without any evidence of pitting or localized corrosion.

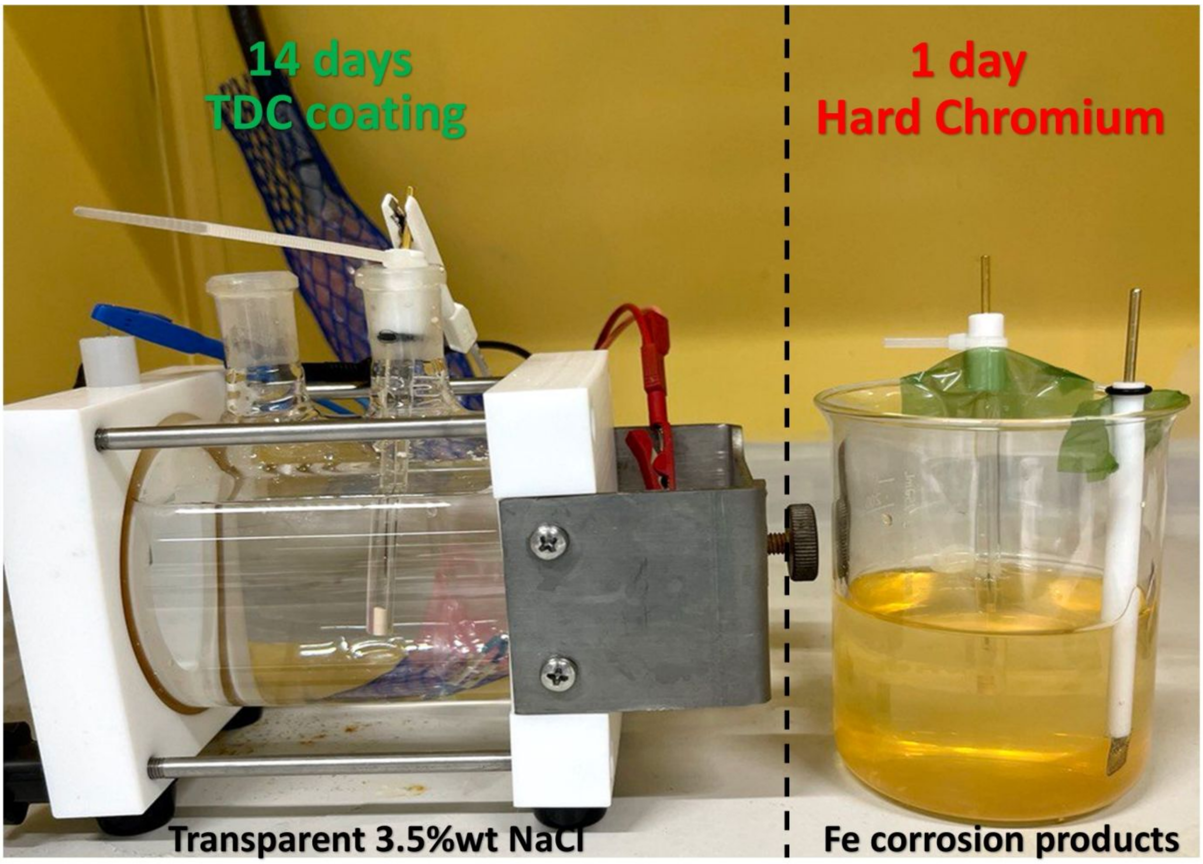

Figure S9. Optical macrograph images of hard chromium and TDC coatings during EIS monitoring (e.g., free exposure) reveal significant differences in corrosion resistance. After 14 days of exposure, the TDC coating showed no signs of corrosion or contamination from the 3.5% wt NaCl solution, remaining highly transparent. In contrast, the hard chromium coating exhibited substantial corrosion after just 1 day of exposure, resulting in a significant release of Fe metal ions into the solution.

Table S1. The binding energy of Cr 2p at three different exposed times including fresh surface, 0.4V polarized 3.5% NaCl, and 24h free exposure in 3.5% NaCl.

**Exposed samples**	**Cr (eV)**	**Cr(III)-1 (eV)**	**Cr(III)-2 (eV)**	**Cr(III)-3 (eV)**	**$Cr(OH)_3$ (eV)**	**Cr(III)-4(eV)**	**CrO_3**
Fresh surface	573.39	574.59	575.46	576.20	577.05	578.03	579.05
0.4V polarized	573.57	574.63	575.54	576.40	577.05	578.01	579.10
24h exposure	574.10	574.84	576.07	577.02	577.93	578.83	579.81
14 days exposure	573.15	574.24	575.77	576.67	577.78	579.03	580.05

Table S2. Fitting data obtained from EIS measurements in Figure 4 using an R(RCPE) equivalent electrical circuit.

Monitoring time (h)	R_s ($\Omega\cdot cm^2$)	CPE_{dl} ($\mu F\cdot cm^{-2}s^{n-1}$)	n_{dl}	R_{ct} ($M\Omega\cdot cm^2$)	$CPE_{complex\ oxide}$ ($\mu F\cdot cm^{-2}\ s^{n-1}$)	$n_{complex\ oxide}$	$R_{complex\ oxide}$ ($k\Omega\cdot cm^2$)	X^2
**1h**	23.4±0.2	14.6±0.6	0.97±0.01	5.3±0.9	38.9±2.7	0.81±0.009	69.5±3.9	0.0015
**2h**	23.4±0.1	12.7±0.5	0.98±0.01	14.2±1.5	64.9±5.4	0.79±0.01	50.4±3.5	0.0012
**3h**	23.6±0.1	12.6±0.4	0.97±0.01	19.4±1.8	68.6±6.8	0.79±0.01	46.5±3.8	0.0012
**4h**	23.3±0.1	12.5±0.4	0.97±0.01	21.3±1.6	69.7±5.2	0.78±0.01	43.9±2.9	0.0011
**5h**	23.4±0.1	12.5±0.3	0.97±0.01	22.7±1.9	71.9±5.7	0.78±0.01	41.9±2.2	0.0012
**6h**	23.3±0.1	12.4±0.4	0.96±0.01	23.4±2.2	78.0±5.9	0.78±0.02	37.1±3.8	0.0012
**8h**	23.4±0.1	12.3±0.5	0.96±0.01	23.6±1.3	82.1±6.4	0.78±0.02	32.7±3.7	0.0012
**10h**	23.5±0.1	12.4±0.4	0.96±0.01	24.4±1.6	83.6±6.7	0.78±0.02	31.4±3.5	0.0012
**14h**	23.5±0.4	12.4±0.4	0.96±0.01	24.2±1.4	82.4±6.8	0.78±0.02	30.9±2.7	0.0012
**18h**	23.6±0.1	12.4±0.4	0.96±0.01	23.9±1.9	81.8±6.5	0.78±0.01	30.6±2.2	0.0012
**24h**	23.5±0.4	12.5±0.4	0.96±0.01	22.6±1.5	80.7±5.4	0.78±0.01	31.1±2.5	0.0012
